# Developing an Undergraduate Applied Learning Experience

**DOI:** 10.3389/fpubh.2015.00002

**Published:** 2015-02-13

**Authors:** Denise C. Nelson-Hurwitz, Michelle Tagorda

**Affiliations:** ^1^Office of Public Health Studies, University of Hawai‘i at Mānoa, Honolulu, HI, USA

**Keywords:** bachelors of public health, curriculum, undergraduate capstone, undergraduate public health education, undergraduate research

## Abstract

To foster student development, critical thinking, and application skills among public health students at the University of Hawai‘i at Mānoa, a three-course capstone series was developed as a key component of the public health Bachelor of Arts degree program. Over the course of 1.5 academic years students are actively involved in developing an interdisciplinary project proposal, then executing and presenting an independent, supervised, applied learning project. In the first course, students are introduced to a diverse range of public health projects and methods while working to develop their own project proposal – the foundation for the applied learning experience. The project execution course is designed to allow students to execute their proposed applied learning projects. This experience focuses on the application and integration of public health knowledge, skills, and practice acquired during the bachelor’s degree course of study. Finally, students will be involved in reflecting on, finalizing, and sharing their completed projects in an undergraduate capstone seminar. Through implementation of this series, the program hopes to provide students with the opportunity to actively apply academic skills to real-world application.

## Background

It has been recommended by several entities that undergraduate programs, particularly those focused on public health, include opportunities for integrative and experiential learning (1–4). Experts have also recommended bachelor degree programs specifically include a capstone experience, promote inquiry-based learning, and better involve students in research activities, specifically at research universities^1^ (5–7). Furthermore, the Institute of Medicine (8) recommended public health as an essential part of training for citizens – a proposal supported by the Association of American Colleges and Universities (6) as well as several other academic organizations (3).

During the development of the Bachelor of Arts degree in Public Health at the University of Hawai‘i at Mānoa (a research university and land-, sea-, and space-grant institution), a three-course integrative learning experience capstone was created so as to provide students with an opportunity to apply course knowledge and skills to a real-world issue, develop a passion for a topic of personal interest, and gain experience working with collaborators (possibly in the community). This capstone series has been named the applied learning experience.

### Development of capstone series

The three-course applied learning experience was initially developed and proposed by a departmental committee comprises faculty, staff, and a student representative, formed to develop the B.A. Public Health program for the University of Hawai‘i at Mānoa (UHM). The need for a capstone experience was supported during preliminary meetings with both local community members, many of who would be potential employers of our graduates, and key on-campus stakeholders. The UHM Honors Program initially inspired the three-course progression, where students are guided through development of a project proposal in one semester then execute the project in subsequent semesters. The process was further refined to reflect the needs of students, faculty, and community members as identified from past experiences with the Master of Public Health (MPH) practicum and subsequent MPH capstone course.

## Applied Learning Experience Program Structure

The intent of the culminating experience in the B.A. program is to provide students the opportunity to actively apply classroom knowledge and associated skills to real-world application in the public health field. This is facilitated by a three-course series required for graduation with the B.A. Public Health degree: PH480 Application of Public Health Principles in Research and Practice, PH485 Applied Learning Experience, and PH489 Public Health Undergraduate Capstone Seminar.

Student project selections may have either a research or practice-based focus, depending on the preference of the individual student, as well as their post-graduation plans. For example, a student interested in employment with a non-profit organization following graduation may prefer a service-learning oriented experience with a local non-profit, whereas a student applying for a graduate program may be more interested in working with, or designing their own, research project. Examples of selected student topics are included in Table 1.

**Table 1:** **Examples of selected applied learning experience projects**.

Research-based project examples	Service-learning oriented project examples
Key factors in obstetric decision-making among women with limited English proficiency	Exploring methods to reduce substance abuse in Hawai‘i (partnership with Gregory House Programs Hawai‘i)
Evaluating the effectiveness of home visiting on maternal-child health	Increasing awareness of kidney disease in Hawai‘i (partnership with National Kidney Foundation – Hawai‘i Chapter)
Methods of using social media to promote physical activity among college students	Enhancing nutrition education and healthy eating in Hawai‘i Public Schools (partnership with Hawai‘i Department of Health and Hawai‘i Department of Education)
Developing an approach to improving access to health care among Oahu’s homeless population	Health, fitness, and academic achievement among youth in Hawai‘i Public Schools (partnership with Hawai‘i Department of Health and Hawai‘i Department of Education)

Throughout the applied learning experience each course instructor evaluates students on performance and competence through class assignments and class activities. The student’s selected, and approved mentor or advisor additionally assesses the execution of the applied learning experience during PH485. Additional Public Health departmental faculty members, other University of Hawai‘i at Mānoa faculty, or approved community experts are encouraged to serve as advisors and mentors to specific applied learning experience projects, as appropriate to the student-selected topics.

### Undergraduate public health summit

The Undergraduate Public Health Summit is a semi-annual, open, public forum, and a vital opportunity for students to interact with faculty (in public health and other disciplines), program alumni, and other community resources. Students participate in the Undergraduate Public Health Summit twice during their capstone series. First, they are involved in presenting their applied learning experience project proposals as poster presentations within the last 2 weeks of PH480. Students are also required to present their final applied learning experience projects (experiences and any results or analyses) as both poster and oral presentations during the last weeks of the PH489 capstone seminar. This experience provides students with practice presenting their projects and outcomes in a professional format and both interacting and receiving feedback from a range of forum participants.

### PH480: application of public health principles in research and practice

This course is typically taken during the first semester of a student’s third (junior) year of the Bachelor of Arts degree program, and is the students’ first introduction to the applied learning experience. The purpose of PH 480 is to prepare students for an independent, supervised, integrated learning project to be performed as part of the public health undergraduate capstone experience. The course begins with career counseling and post-graduation preparation in a group setting. By focusing on both short- and long-term personal objectives students are better able to create an experience that will support their future goals. This course also exposes students to common research tools and practices, including writing a literature review, creating a written and oral project proposal, designing a poster for public presentation, seeking external funding, ethics education, and working with an Institutional Review Board.

PH480 additionally introduces students to a diverse range of public health projects and associated methods. Throughout the semester, students are exposed to short, 10-min research and practice profiles called “Public Health in Action Profiles.” Profiles are presented by program faculty as well as community partners and provide a brief overview of current public health projects, what responsibilities the presenter’s specific role entails. Profiles are intended to provide undergraduate students with ideas of what public health research looks like in practice and also provide inspiration as students work toward developing their own applied learning experience proposals. For some students, meeting faculty and community partners during presentations also serves as a valuable networking opportunity. Student feedback to Public Health in Action Profiles has been overwhelmingly positive.

At the conclusion of PH480, students will have a written proposal for an applied learning experience project, as well as experience presenting their project proposals at an open, public forum – the Undergraduate Public Health Summit.

### Experience proposal guidelines

Essential components of experience proposals include (1) project objective, (2) project abstract, (3) significance of study or experience, (4) background and literature review, (5) proposed research or experience methodology, (6) expected deliverables, outcomes, or results, (7) proposed collaborators, partners, and next steps, and (8) references. Optional components include an itemized budget, estimate of costs, description of host organization or host research project, and Institutional Review Board application, as appropriate to the specific project.

These components are written in draft form and submitted throughout the semester (roughly four months) of PH480. Feedback is provided for each section, and the final versions of individual components are compiled as a complete project proposal submitted as the final product of the PH480 course. Generally, completed proposals range from 8 to 12 pages (excluding references). Additional research tools developed (e.g., a proposed survey or qualitative research tools) are included as appendices as appropriate.

### PH485: public health applied learning experience

Based on each student’s individual applied learning project proposal, developed during PH480, students are involved in independent research projects under the supervision of both a course instructor and an appropriate, approved project mentor or advisor. Mentors and advisors may be university faculty or approved community experts in a student’s identified research or practice area.

The applied learning experiences take place over the course of a full semester, and involve roughly 100–125 hours of project execution. Students who are executing research-based projects primarily spend their time conducting a more thorough literature review and, in most cases, working to collect data and/or conduct supervised data analyses. Students with practice-focused projects spend the majority of their specified time actively working within an organization under a service-learning format.

Finally, students will be involved in reflecting on, finalizing, and presenting their completed applied learning experience projects throughout an Undergraduate Capstone Seminar (PH489).

### PH489: public health undergraduate capstone seminar

The Public Health Undergraduate Capstone Seminar is taken near the completion of the B.A. degree program. It focuses on integration of public health knowledge, skills, and practice acquired during the course of study. Students will be involved in assessing their level of achievement of educational degree objectives, develop professional goals, and reflect on, finalize, and present their applied learning experience projects. This course also summarizes key content and skills applied throughout the B.A. public health degree program, prepares students for a higher level of learning (either in graduate school or as a working professional), and addresses pragmatic post-graduation skills (e.g., resume/C.V. writing and career planning). Key deliverables following completion of this course include an applied learning reflection paper, a final applied learning experience report, and a final oral and poster presentation delivered at the Undergraduate Public Health Summit.

### Culminating experience evidence

The instructor of the PH489 course and the Undergraduate Program Chair make the final assessment of the undergraduate capstone experience jointly. The undergraduate culminating expectations include the following:

- Address a key issue, concern, or research problem related to the field of public health;
- Apply knowledge and skills accumulated through public health course work to address the selected issue/problem;
- Demonstrate integration and practical application of public health concepts; and
- Demonstrate appropriate written and oral communication skills.

Concrete evidence of successful completion of culminating experience include the following:

- Written project proposal for applied learning experience;
- Poster presentation of applied learning experience project proposal at Undergraduate Public Health Summit;
- Completion and submission of signed mentor/advisor agreement form;
- Completion and submission of final applied learning experience assessment of advisor or mentor;
- Completion and submission of applied learning reflection paper;
- Completion and submission of final applied learning project report; and
- Oral presentation of final applied learning project at Undergraduate Public Health Summit.

Students must also complete and obtain passing grades (above “C−”) in all required and elective coursework associated with the B.A. in Public Health degree as evaluated by faculty instructors indicating mastery of the content and competency.

## Discussion

The applied learning experience serves as a capstone to the B.A. Public Health degree program at the University of Hawai‘i at Mānoa. Through a three-course series, students engage in inquiry-based learning centered on a topic of interest of their choosing. As previously discussed, there are two potential tracks for Applied Learning Experience projects – research or practice based, so as to allow students to select which type of experience would be most suitable to their academic needs and future plans. Both tracks provide an opportunity for integrative and experiential learning through slightly variable approaches. The research-based track exposes students to the inner workings of academic investigation, whereas the practice-based track provides real-world work experience. Both tracks include continuous application of critical thinking skills and student reflection, particularly throughout the execution of the applied learning experience in keeping with a service-learning framework.

Preliminary feedback from B.A. students who are currently participating in the applied learning experience has been very positive overall. The primary adjustment made based on student feedback has been to place more emphasis on advising of students preparing for the undergraduate culminating experience during mandatory advising session. The intent of this emphasis would be to help students begin thinking about possible projects and to increase awareness of the three-course series in the context of their full undergraduate degree program.

Through the applied learning experience, this program hopes to better prepare undergraduate public health students for both public health practices in the workforce, improve transition into graduate programs, and more broadly, fully embrace application of the educated citizen framework (8).

## Conflict of Interest Statement

The authors declare that the research was conducted in the absence of any commercial or financial relationships that could be construed as a potential conflict of interest.

## Footnotes

^1^Council on Undergraduate Research. Faculty-Undergraduate Collaborative Research and Publishing (2003).
